# A rapid and visual dual LAMP-LFD assay for on-site simultaneous detection of influenza A virus (H1N1) and respiratory syncytial virus (RSV)

**DOI:** 10.3389/fmicb.2025.1678396

**Published:** 2025-11-10

**Authors:** Alang Zhang, Xingyu Lan, Hengxuan Zhou, Ting Zhou, Zhihua Xu, Xinyu Cheng, Chenxi Guo, Mingjie Wei, Jiahui Wu, Feng Shi

**Affiliations:** 1 College of Life Sciences, Shihezi University, Shihezi, China; 2 The First Division Hospital of Xinjiang Production and Construction Corps, Akesu, China

**Keywords:** double detection, respiratory viruses, loop-mediated isothermal amplification, lateral flow device, visual detection

## Abstract

Respiratory tract infections caused by influenza A virus (IAV) and respiratory syncytial virus (RSV) are a major global health burden. To overcome the limitations of existing diagnostic techniques for timely detection (point-of-care testing), this study developed a dual rapid visual detection method based on loop-mediated isothermal amplification (LAMP) and lateral flow device (LFD) technologies for the simultaneous detection of H1N1 influenza virus and RSV. This method uses a dual-labeled probe system (H1N1: digoxigenin/biotin; RSV: 6-carboxyfluorescein/biotin) combined with a two-color latex microsphere signal system that enables the intuitive visual interpretation of multiple detection results. Compared with traditional nucleic acid detection, the entire detection process was completed within 40 min at a constant temperature of 63 °C and the operation was simple. After optimization, the method showed good sensitivity and specificity. The limit of detection for H1N1 IAV and RSV was as low as 7.78 × 10^3^ copies/mL and 1.29 × 10^2^ copies/mL, respectively, and no cross-reaction occurred with six common non-target pathogens. Clinical sample verification showed that the consistency between the detection and RT-qPCR results was 96.83%, which was significantly better than traditional antigen detection. The dual LAMP-LFD detection method established in this study provides an efficient solution for rapid on-site, synchronous, and visual screening in resource-limited environments and has important clinical application value.

## 1 Introduction

Respiratory infectious diseases, especially those caused by influenza A virus and respiratory syncytial virus (RSV) (Avendaño Carvajal and Perret Pérez, 2020), pose a major threat to global human health and are a major cause of significant public health burdens worldwide (GBD 2017 Influenza Collaborators, 2019). They not only become the main pathogenic factors in the outbreak of seasonal influenza, but also cause a variety of upper and lower respiratory tract infections in other cases. Rapid diagnosis of disease caused by these pathogens is key to achieving timely clinical intervention, effectively blocking the transmission chain and controlling outbreaks (Alahmari et al., 2024). As the main pathogenic factor of influenza, the influenza A virus exhibits strong variability, can cause large-scale influenza epidemics, and has potential global pandemic risks (Wu et al., 2023). More than 3 million severe cases of influenza A virus infection and more than 300,000 related deaths are reported each year worldwide. RSV is a major viral pathogen that causes acute lower respiratory tract infections in both infants and elderly individuals (Guo L. et al., 2024). RSV infection not only causes mild upper respiratory symptoms but can also lead to severe pneumonia and bronchitis, especially in immunocompromised individuals who are more likely to deteriorate (Pandya et al., 2019; Chadha et al., 2020). According to global epidemiological estimates, respiratory syncytial virus (RSV) was associated with over 33 million cases of infection worldwide in 2019. Notably, RSV-attributable acute lower respiratory infections caused 101,400 deaths in children under 5 years of age, among whom 45,700 were infants younger than 6 months (Li et al., 2022). Therefore, the early diagnosis of influenza A virus and RSV is important for clinical management.

Presently, detection methods for respiratory pathogens mainly include virus isolation and culture (Nijhuis et al., 2017), direct or indirect immunofluorescence assays (IFAs) (Liolios et al., 2001), and nucleic acid detection (Bruning et al., 2017). Virus isolation and culture, which are a classical method for virus identification, are limited by time-consuming, cumbersome operation steps and insufficient standardization, which make it difficult to meet the needs of on-site emergency detection (De et al., 2022). Although the immunodetection method is fast and efficient, its sensitivity and specificity for virus detection are relatively low due to the continuous antigen drift caused by the high frequency mutation of the conserved sequence of the influenza virus (da Mesquita et al., 2017). Nucleic acid detection technology has become an important method for detecting respiratory viruses because of its high sensitivity and specificity. Real-time quantitative polymerase chain reaction (RT-qPCR) is the gold standard for detecting respiratory viruses in most hospitals. However, the dependence of this method on expensive instruments and professional operators limits its application to limited resources and onsite environments (Ravina et al., 2020).

With the development of molecular biology technology, loop-mediated isothermal amplification (LAMP) (Notomi, 2000; Nagamine et al., 2002), a new nucleic acid amplification technology, has become an effective tool for the rapid detection of pathogens owing to its isothermal amplification characteristics, rapid response, and requirement for simple equipment (Iuliano et al., 2018). Its core advantages are its significantly improved specificity and fault tolerance, which are achieved through the use of four to six primers to identify six to eight regions of the target gene; amplification remains effective even in the presence of individual mutations in the target sequence, which is critical for RNA viruses with high mutation rates (e.g., influenza A virus and RSV) (Kautz and Forrester, 2018; Shishir et al., 2023). The LAMP reaction achieves exponential enrichment of target DNA/RNA sequences under constant temperature conditions, greatly improving the timeliness and accuracy of detection. Additionally, only basic temperature control equipment is required, which is suitable for rapid on-site detection. Although detection methods for LAMP amplification products are diverse, they have technical limitations. Real-time turbidity monitoring tracks amplification by detecting turbidity changes caused by the precipitation of magnesium pyrophosphate, a by-product of LAMP, or by visualizing signal accumulation using fluorescent dye/probe labeling. However, these methods rely on precision instruments such as turbidimeters and fluorescence detectors. Nucleic acid dyes such as SYBR Green allow for naked-eye interpretation by intercalating double-stranded DNA to undergo color conversion. Although easy to perform, it has a high risk of producing false positives and a low level of detection specificity owing to non-specific binding to all double-stranded structures (including non-target sequences) (Kim et al., 2023). Agarose gel electrophoresis (AGE), a conventional method, involves separation of DNA fragments according to size via electrophoresis and assessment through ladder-like strips presented by nucleic acid staining. However, nonspecific amplification products (e.g., primer dimers) can lead to false-positive bands and can easily cause nucleic acid aerosol pollution (Mannier and Yoon, 2022; Yu et al., 2022). Although these detection methods are fast and sensitive, they cannot achieve multiple visual detections, limiting their application in the diagnosis of acquired respiratory viruses in resource-poor areas. As a simple, rapid, and cost-effective detection platform, lateral flow immunoassay [lateral flow device (LFD)] has been widely used in various immunological diagnostic fields (Kakkar et al., 2024). This technology is based on the principle of specific antigen-antibody binding, and the target substance in the sample is intercepted by the capture antibody fixed on the nitrocellulose (NC) membrane to form a visible detection line (T line) (Li et al., 2023). When applied to nucleic acid detection, LFD utilizes oligonucleotide probes to specifically hybridize with biotin-labeled LAMP amplification products and then achieves signal transduction and visual output through the biotin-streptavidin system (Cheng et al., 2024). This technology offers the advantages of simple operation, low cost, and high detection speed and has therefore, been widely used for the rapid detection of respiratory pathogens.

To meet the urgent need for rapid on-site diagnosis [point-of-care testing (POCT)] of influenza A virus and RSV, this study aimed to develop a visual method for dual virus detection with single amplification based on LAMP and lateral flow test strip technologies. This method is simple, rapid, and sensitive, and can be used as a field screening tool to provide a new strategy for the early diagnosis, prevention, and control of respiratory viral infections.

## 2 Materials and methods

### 2.1 Materials and reagents

Bst 2.0 WarmStart DNA Polymerase, WarmStart RTx Reverse Transcriptase, 10 × Isothermal Amplification Buffer, MgSO_4_, and LAMP Fluorescent Dye were purchased from New England Biolabs (Ipswich, MA, USA). Heat-labile UDG enzyme, dNTPs and the plasmid extraction kit were purchased from Vazyme Biotech Co., Ltd (Nanjing, China). Anti-fluorescein isothiocyanate (FITC) rabbit polyclonal antibody was provided by Bioengineering Co., Ltd (Shanghai, China). Mouse anti-digoxin monoclonal antibody, Chicken Yolk Immunoglobulin (IgY), and goat anti-chicken IgY antibody were acquired from Biodragon Technology Co., Ltd (Suzhou, China). Nitrocellulose (NC) membranes, sample pads, and conjugate pads were sourced from Millipore Sigma (Darmstadt, Germany). All primers, probes, and standard plasmids were synthesized by Sangon Biotech Co., Ltd (Shanghai, China). Other chemical reagents were purchased from McLean Biochemical Technology Co., Ltd (Shanghai, China).

### 2.2 Sample and DNA preparation

According to a previous study by Lin et al. (2021), the highly conserved regions were selected as the target sequences using the GenBank nucleic acid sequence database established by the National Center for Biotechnology Information (NCBI).^1^ H1NI influenza A virus nucleic acid hemagglutinin (HA) (GenBank accession number NC_007366.1) and RSV fusion (F) protein (GenBank accession number NC_001803.1) gene sequences were selected as targets for this method. DNAMAN was used for multiple sequence alignments of different subtypes of the same virus, and relatively conserved gene fragments were identified for NCBI BLAST verification to ensure the specificity of the selected sequence. The verified gene fragments of the two respiratory viruses were inserted into plasmid PUC57 to prepare the plasmid standard, which was synthesized by Sangon Biotech Co., Ltd (Shanghai, China).

### 2.3 Primer and probe design for LAMP detection of respiratory pathogens

**FIGURE 1:**
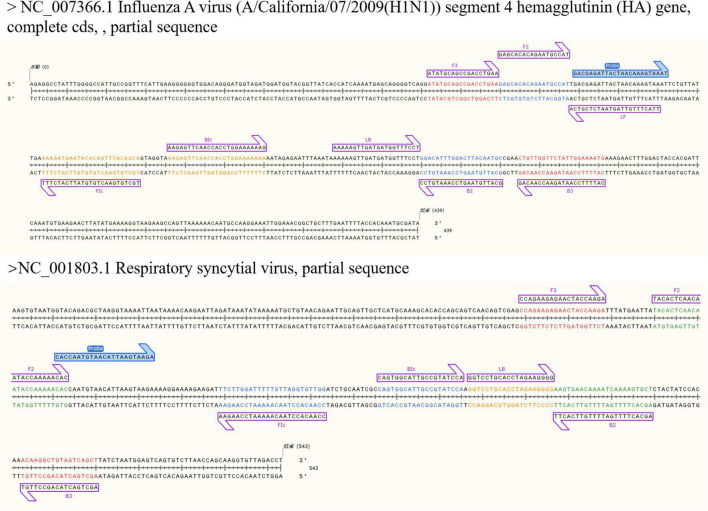
Conserved sequences (partial) of HA gene of influenza A virus H1N1 subtype and F gene of respiratory syncytial virus and LAMP primers (highlighted) for construction, including outer primers (F3/B3), inner primers (FIP/BIP), loop primers (LF/LB) and special probes. The internal primer FIP/BIP is composed of FIc and Bic and the complementary sequences of F2 and B2.

The following LAMP primers were designed using Primer Explorer V5^2^ : two external primers (F3 and B3), two internal primers (FIP and BIP), and loop primers (LF and LB). NUPACK software^3^ was used to predict the specificity between primers and reduce the influence of primer dimers, which is not conducive to LAMP amplification. Specific probes were designed for the conserved sequences. The 5′ of the FIPs was modified with biotin, and the specific probes were labeled with digoxigenin (DIG) and 6′ carboxyfluorescein (FAM) for application in the LFD system. All oligonucleotide sequences and their physical locations are shown in Table 1 and Figure 1.

**TABLE 1:** The specific primers and probes designed for H1N1 and RSV detection.

Virus	Gene	Primer	Sequence 5′-3′
H1N1	HA	F3	ATATGCAGCCGACCTGAA
		B3	CATTTTCCAATAGAACCAACAG
		FIP	Biotin-TGCTGTGAACTGTGTATTCATCTTTGAGCACACAGAATGCCAT
		BIP	AAGAGTTCAACCACCTGGAAAAAAGGCATTGTAAGTCCAAATGTCC
		LF	TTACTTTGTTAGTAATCTCGTCA
		LB	AAAAAGTTGATGATGGTTTCCT
		Probe for LAMP-LFD	DIG-GACGAGATTACTAACAAAGTAAAT
RSV	F	F3	CCAGAAGAGAACTACCAAGA
		B3	AGCTGACTACAGCCTTGT
		FIP	Biotin-CCAACACCTAACAAAAATCCAAGAATACACTCAACAATACCAAAAACAC
		BIP	CAGTGGCATTGCCGTATCCAAGCACTTTTGATTTTGTTCACTT
		LB	GGTCCTGCACCTAGAAGGGG
		Probe for LAMP-LFD	6′FAM-CACCAATGTAACATTAAGTAAGA

### 2.4 LAMP reaction system

The total mixture volume of the LAMP reaction system was 25 μL, including 2.5 μL of 10 × isothermal amplification buffer, 1 μ of mixed enzyme solution (including 8 U of Bst 2.0 DNA Polymerase and WarmStart RTx Reverse Transcriptase), 6 mM MgSO4, 1.4 mM dNTP mix, 1.6 μM FIP/BIP, 0.2 μM F3/B3 primer, 0.4 μM Loop F/B Primer, and 2 μL of the target DNA template. In addition, 1 U of thermally unstable uracil-DNA glycosylase and uracil triphosphate at a final concentration of 0.7 mM were added to the reaction system to reduce the risk of contamination. Each reaction was carried out in a 0.2 mL microcentrifuge tube, and the temperature and time were controlled using a metal bath. The NEB fluorescent dye was added to the real-time fluorescent LAMP detection system, and detection was performed on a LightCycler^®^ 480 II real-time fluorescence quantitative PCR instrument (Roche, Basel, Switzerland) and Bosch Line Gene 9660 Plus fluorescence quantitative PCR instrument (Bioer, Hangzhou, China). The LAMP reaction was performed at 63 °C for 45 min and terminated at 85 °C for 5 min.

### 2.5 LAMP reaction system optimization

To determine the optimal temperature for real-time fluorescent LAMP detection of the two target respiratory viruses, the reaction system described in section “2.4 LAMP reaction system” was employed. Under identical experimental conditions, real-time fluorescent LAMP reactions were conducted at six different temperatures (55 °C, 57 °C, 60 °C, 63 °C, 65 °C, 67 °C) for 45 min using a real-time PCR instrument, with fluorescence signal acquisition performed every minute. Based on the identified optimal reaction temperature, the ratio of inner to outer primers (2:1, 4:1, 8:1, 16:1, 32:1) was optimized in a 25 μL reaction volume for real-time fluorescent LAMP amplification. Subsequently, optimization of Mg^2+^ concentration (2, 4, 6, 8, and 10 mM), and Bst DNA polymerase activity (4.0, 6.0, 8.0, 12.0, and 16.0 U) was performed. All optimization reactions were incubated at the constant optimal temperature for 45 min, and fluorescence signals were collected at one-minute intervals. The optimal reaction system configuration was determined based on the analysis of amplification curves.

### 2.6 Development of the LAMP-LFD

**FIGURE 2:**
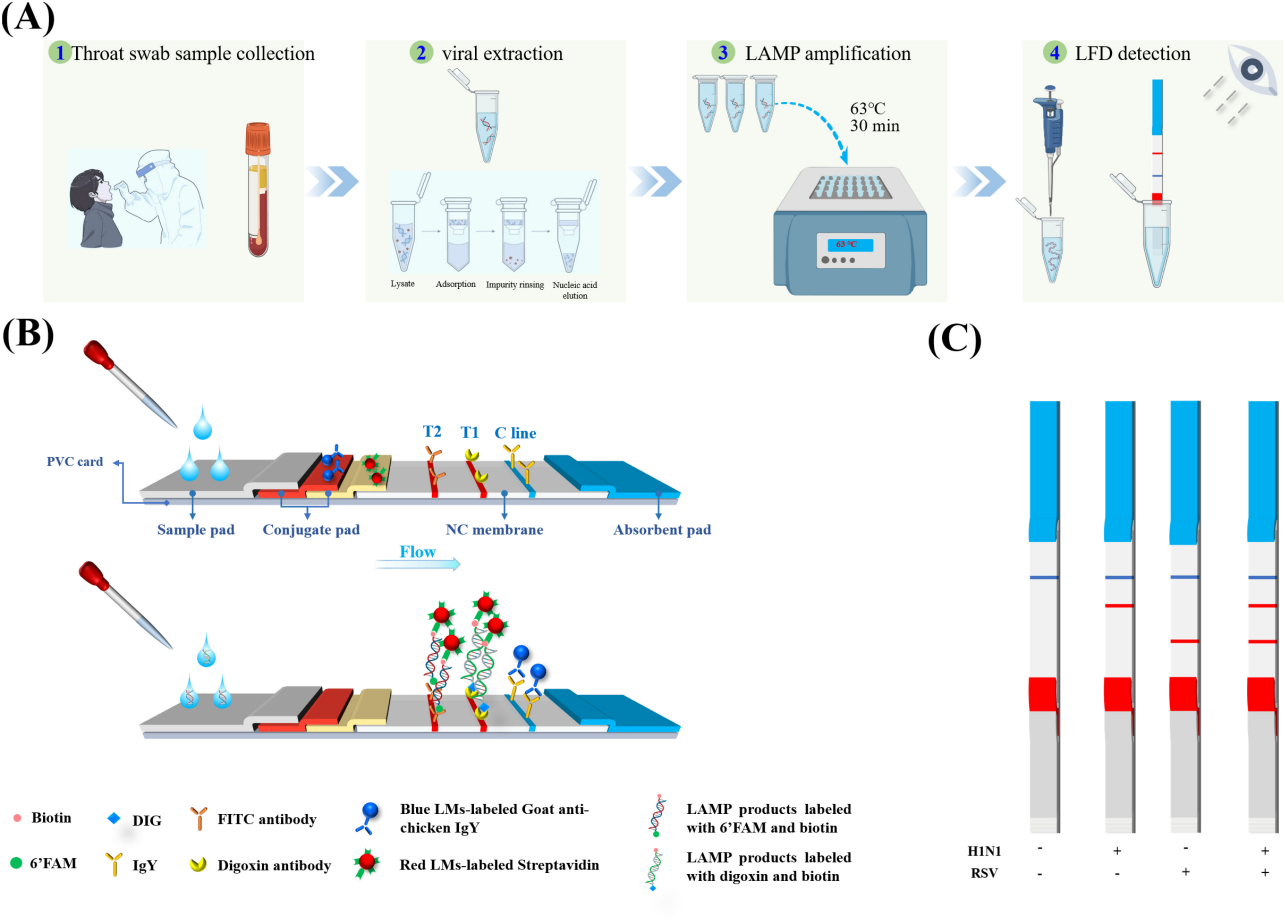
Simultaneous detection of H1N1 and RSV by LAMP-LFD. **(A)** Schematic diagram of detection process: showing the whole process from sample processing to LAMP amplification and visual reading of lateral flow detection (LFD). **(B)** Schematic of the LFD detection principle: based on dual-labeled probe system and dual-color latex microsphere signal system, the structural design of visual interpretation of multiple targets is realized **(C)** Interpretation of four signal outputs of LAMP-LFD method.

The lateral flow dipstick was composed of a glass fiber membrane (sample pad), conjugate pad, nitrocellulose membrane, absorbent pad, and polyvinyl chloride backing card. Blue latex microspheres labeled with chicken IgY and red latex microspheres labeled with streptavidin were mixed at a 1:2 ratio and sprayed onto the binding pad. On the NC membrane, detection lines T1 and T2 and the quality control line (C line) were sprayed with mouse anti-digoxin monoclonal antibody, rabbit polyclonal antibody against fluorescein isothiocyanate (FITC), and goat anti-chicken IgY antibody, respectively. After drying at 37 °C for 12 h, the assembled test strip was cut into 3 mm-wide strips and stored under dry conditions. A schematic diagram illustrating the overview and detection workflow of the LAMP-LFD method is provided in Figure 2A. We have previously described the preparation and assembly of test strips (Zhu et al., 2019).

The chromogenic mechanism of the test strip is based on an immunological double-antibody sandwich structure (Figure 2B). Presence of the LAMP product in the sample enables the specific binding of the biotin-modified amplicon to streptavidin-labeled red latex microspheres on the binding pad to form two ternary complexes (FAM or DIG-nucleic acid-streptavidin-labeled red latex microspheres). Through capillary migration, the IgY-labeled blue latex microspheres and the complexes formed in the sample pad pass through the T1/T2/C line on the NC membrane. The FITC-DNA-biotin-red and DIG-DNA-biotin-red latex microsphere antibody ternary complexes are captured by the anti-FITC and anti-DIG antibody on the T1 and T2 detection lines, respectively. Both cause the red latex microspheres to aggregate and form a red band. Meanwhile, IgY-labeled blue latex microspheres are only captured by the goat anti-chicken IgY antibody on the C line, forming a blue band. Therefore, a single blue control line indicates negativity. A blue C line and red T1 line indicate H1N1 positivity, while a blue C line and red T2 line indicate RSV positivity (Figure 2C), and the blue band was used to verify the effectiveness of the chromatography process.

### 2.7 Optimization of the LAMP-LFD assay

To verify the feasibility of LAMP-LFD detection and determine the optimal reaction time, a series of control samples was set up: a positive reaction system containing the influenza A virus H1N1 and RSV targets, no-template control (NTC) and mixed negative samples, and H1N1/RSV single positive/mixed positive cross-reaction control. The LAMP amplification products were incubated for varying durations (0, 10, 20, 30, 40, 50, and 60 min) and Different probe concentrations (2 pM to 200 nM). After the preparation of the reaction system is completed, an appropriate amount of paraffin oil is added to cover the liquid surface to further prevent aerosol pollution. After completion of the LAMP reaction, specific probes labeled with DIG and FAM for detecting influenza A virus and RSV, respectively, were added. Each had a concentration of 20 nM and was mixed and incubated at 65 °C for 5 min. The probe hybridized with the amplified DNA target sequence, and the hybridization product was formed with biotin and FAM or biotin and DIG to form a LAMP product that had a double-labeled double-antibody sandwich structure. One microliter of the double-labeled amplification product was mixed with 80 μL of test strip buffer in the enzyme-labeled microplate. The test strip was inserted into the mixed plate, and the results were read after 5–10 min.

### 2.8 Sensitivity and specificity of the LAMP-LFD

The sensitivity of the LAMP-LFD detection method for the two viruses was evaluated using different concentrations of genomic DNA as templates. The template was serially diluted 10 times to make a working concentration range of 7.78 × 10^8^ copies/mL to 7.78 × 10^0^ copies/mL and 1.29 × 10^9^ copies/mL to 1.29 × 10^0^ copies/mL. LAMP was performed using the optimized reaction system and reaction conditions, and the products were detected via AGE and LFD visualization. Each experiment was repeated thrice, and sterile water was used as a negative control. The limit of detection (LOD) was defined as the minimum detectable concentration by the LAMP-LFD method. The specificity of the LAMP-LFD assay was evaluated using DNA and RNA templates from six non-influenza A virus and RSVs: human parainfluenza virus (HPIV), influenza B viruses (IBVs), *Mycoplasma pneumoniae* (MP), coxsackievirus, *Citrobacter freundii*, and *Escherichia coli*. The templates of all strains or standards were extracted using a FastPure^®^ Viral DNA/RNA Mini Kit Pro (Vazyme Biotech Co., Ltd., Nanjing, China) and stored at −80 °C before use.

### 2.9 Validation using real clinical samples

From March 2024 to April 2025, 63 throat swab samples were collected from the First Division Hospital of Xinjiang Production and Construction Corps for the validation study. All samples were clinically confirmed by RT-qPCR, comprising 36 H1N1- or RSV-positive and 27 negative samples. Total RNA of the influenza A virus and RSV was extracted from clinical throat swab buffer using FastPure^®^ Viral DNA / RNA Mini Kit Pro (Vazyme Biotech Co., Ltd.), and 2 μL of RNA template was used for RT-LAMP. Each amplification product was divided into two parts: one aliquot was electrophoresed on a 2% agarose gel to visualize characteristic ladder-like bands while the other was hybridized with H1N1/RSV-specific probes at 65 °C for 5 min, and buffer was added to sample the double-labeled LFD test strip, followed by visual result interpretation after 5 min. All clinical throat swab samples were tested using the RT-LAMP-LFD assay in a blinded manner. Diagnostic performance, including sensitivity, specificity, positive predictive value (PPV), negative predictive value (NPV), and overall agreement rate, was assessed using RT-qPCR as the gold standard.

## 3 Results and analysis

### 3.1 Optimization of LAMP reaction conditions

LAMP reactions for the two respiratory viruses were performed, followed by AGE, which revealed a clear ladder strip, confirming that the amplification was LAMP. However, owing to the ambiguity of the electrophoresis results, the LAMP amplification efficiency could not be clearly expressed. Therefore, a real-time fluorescent LAMP system was constructed to optimize the reaction conditions for the two respiratory viruses.

**FIGURE 3:**
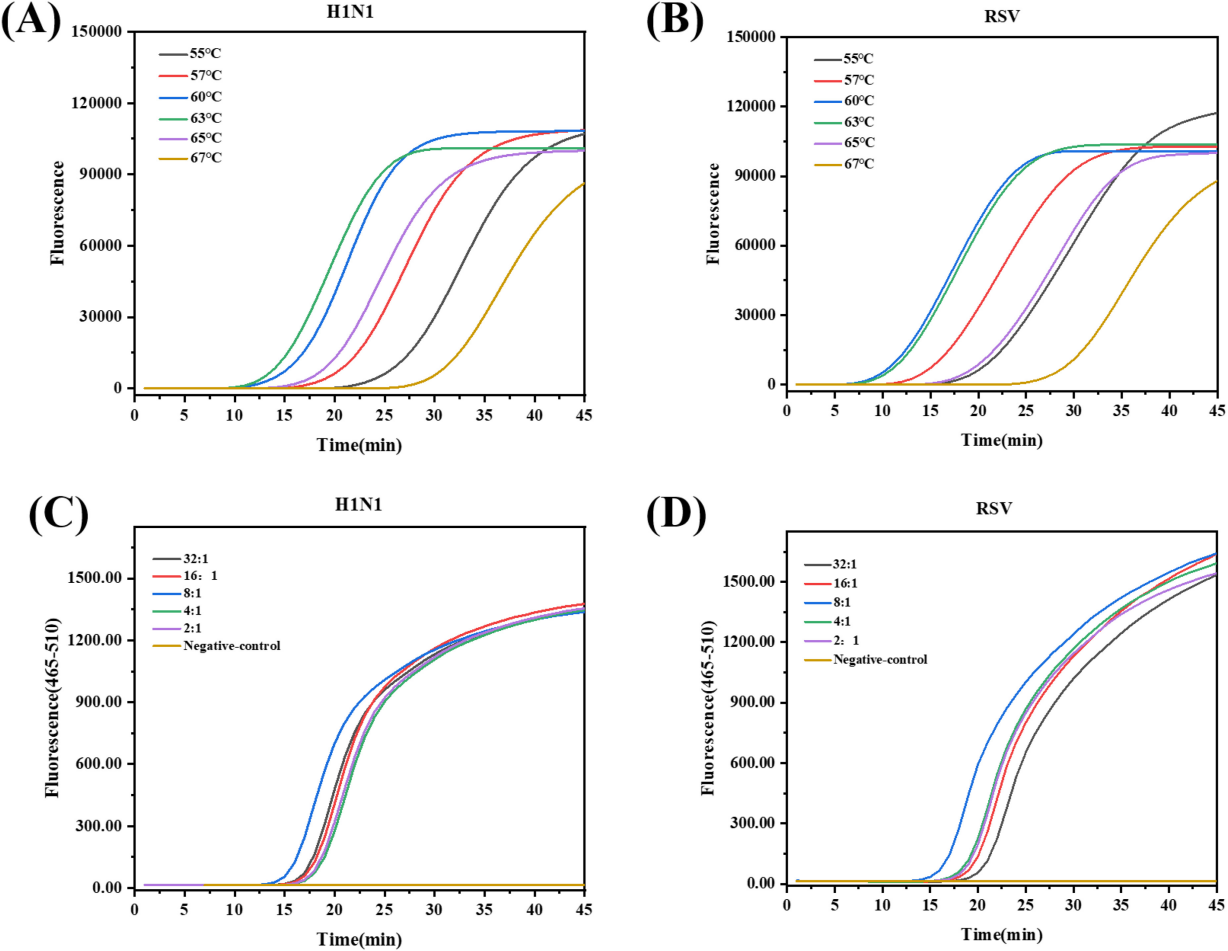
Optimization of reaction temperature and primer ratio for real-time fluorescent LAMP detection of H1N1 and RSV. The real-time fluorescent LAMP fluorescence curve of H1N1 (A)* and RSV (B)* at different incubation temperatures (55 °C, 57 °C, 60 °C, 63 °C, 65 °C, 67 °C). (C)* H1N1 using different proportions of internal primers and external primers LAMP fluorescence curve. (D)* RSV using different proportions of internal primers and external primers LAMP fluorescence curve.*Instruments: **(A,B)** Bosch Line Gene 9660 Plus; **(C,D)** LightCycler^®^ 480 II. Raw fluorescence (RFU) is instrument-dependent and should not be compared directly.

Optimal temperature and time test: Two real-time fluorescent LAMP systems for the respiratory viruses were tested at different temperatures to determine the optimal reaction temperature and time. The fluorescence amplification kinetic curve confirmed that the highest amplification efficiencies were achieved at 63 °C for the H1N1 influenza virus and at 60 °C for RSV (Figures 3A, B). Given that RSV did not significantly impair amplification efficiency at 63 °C, we selected this temperature as the unified LAMP reaction condition for both viruses to streamline the operational procedure of the dual-detection system. Both the respiratory viruses underwent exponential amplification within 30 min and entered the plateau phase. This indicates that the LAMP method only takes 30 min to detect the two respiratory viruses, significantly reducing the detection time compared with that in conventional PCR (usually 90–120 min).

### 3.2 Optimization of the LAMP reaction system

Optimization of the LAMP reaction system is a key step in achieving amplification with high sensitivity and specificity. We optimized the components of LAMP using the optimized reaction temperature and time. The LAMP assay uses multiple primers that work in concert. Non-specific amplification (primer dimers) can occur if the concentration of FIP/BIP concentration is too high, while amplification efficiency is reduced if the concentration is too low (Li et al., 2020). Therefore, balancing primer concentrations is crucial for LAMP amplification. An 8:1 ratio of the inner primer to the outer primer for both viruses resulted in the highest amplification efficiency (Figures 3C, D). Mg^2+^ is an important cofactor of Bst DNA polymerase, which affects primer annealing and non-specific binding during amplification (Martinez-Monge et al., 2022; Dangerfield et al., 2023). Different Mg^2+^ concentrations (2, 4, 6, 8, and 10 mM) were tested. Maximum amplification efficiency was achieved at 4 mM for H1N1 and 6 mM for RSV (Supplementary Figures 1A, B). A unified concentration of 6 mM was adopted for dual detection as it maintained near-optimal efficiency for both targets while simplifying the assay workflow. The Bst enzyme concentration positively correlated with the amplification rate. Examination of the amplification curves indicated that Bst DNA polymerase concentrations of 8 U for H1N1 and 12 U for RSV yielded the highest amplification efficiency in their respective LAMP systems (Supplementary Figures 1C, D). Therefore, a unified concentration of 8 U was adopted for dual detection to maintain near-optimal efficiency while maximizing cost-effectiveness and simplifying the assay workflow.

### 3.3 Establishment of a LAMP-LFD detection method

**FIGURE 4:**
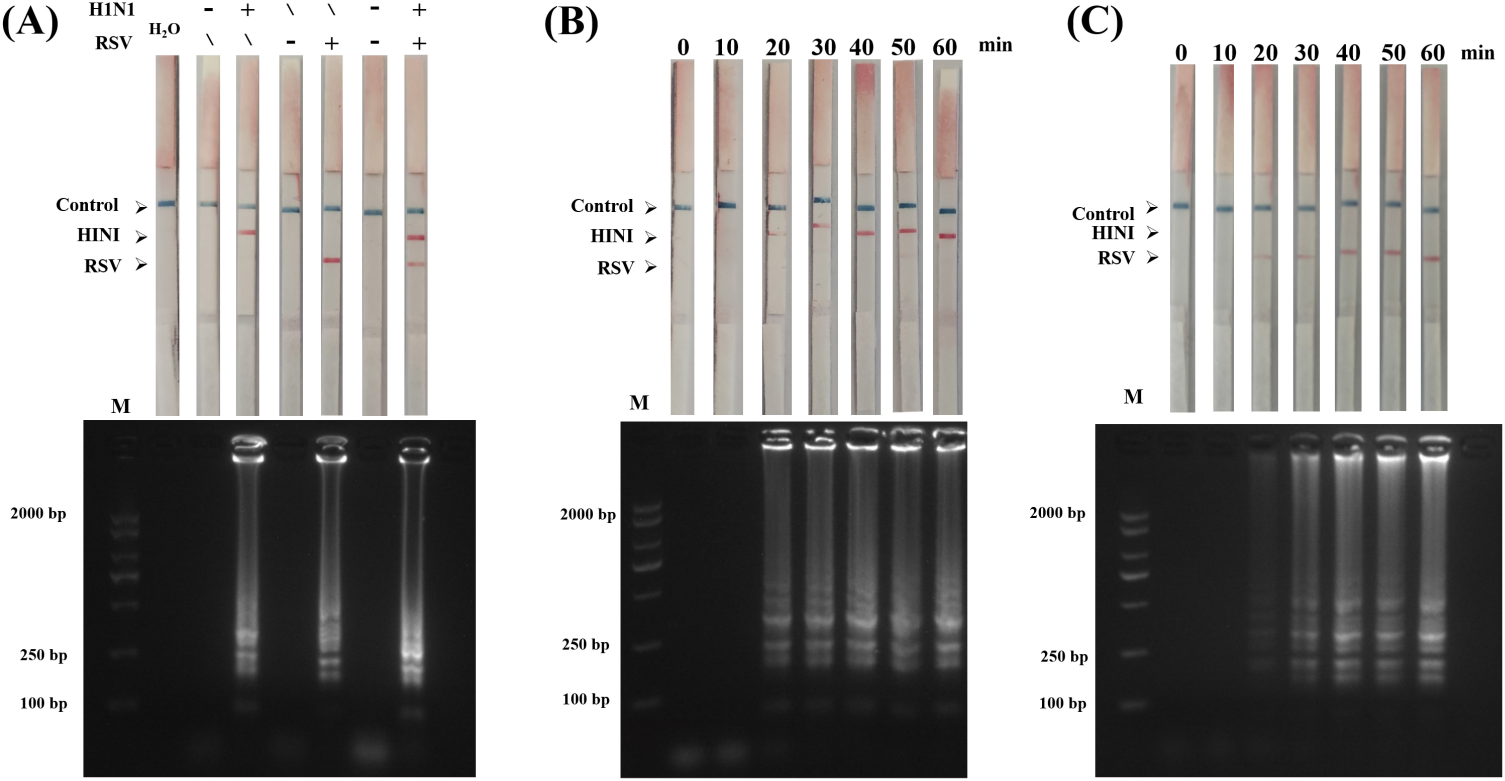
Feasibility of LAMP-LFD detection and optimization of reaction time. **(A)** Feasibility analysis of LAMP-LFD detection : The above figure shows the LFD results of six groups of samples (from left to right: water, H1N1 negative, H1N1 positive, RSV negative, RSV positive, H1N1/RSV mixed negative, mixed positive), and the following figure corresponds to the results of agarose gel electrophoresis (AGE). **(B,C)** H1N1 and RSV were amplified at 63 °C for different time (0, 10, 20, 30, 40, 50, and 60 min). M represents DL2000 Plus DNA Marker (Nanjing, China).

A double-labeled flow immunochromatographic test strip was prepared. To further verify the feasibility of the LAMP-LFD detection method, a series of control samples (influenza A virus H1N1-negative/-positive, RSV-negative/-positive, and H1N1/RSV mixed negative/positive) were used to verify the feasibility of LAMP-LFD detection (Figure 4A). Only the target virus-positive samples showed a clear color on the corresponding detection line (T1 / T2 line), whereas all negative controls showed only the quality control line (C line). These result confirm that no cross-reaction occurred between the primer groups and that the double-labeled probe system can effectively prevent false positives, verifying the feasibility of the method. We also evaluated the probe hybridization concentration (Supplementary Figure 2), and the results indicated that a final probe concentration of 20 nM produced distinct red bands on both the T1 and T2 lines.

To determine the optimal reaction time for LAMP-LFD, we systematically evaluated the detection efficiency at different amplification times (0–60 min). LFD and AGE revealed that (Figures 4B, C) at 20 min of reaction time, T1 and T2 on the test strip appeared as light-colored lines, corresponding to the faint ladder bands that appeared in AGE, indicating that the target can be initially detected at this time. Extension of the reaction time to 30 min resulted in more evident red lines on T1 and T2 and clearer ladder bands in AGE. Therefore, 30 min was the minimum effective amplification time and the H1N1 and RSV detection lines could be clearly interpreted visually. Therefore, the amplification time for all subsequent LAMP-LFD assays was set to 30 min. Combined with the probe hybridization and chromatography time (10 min), the entire detection process was completed within 40 min.

### 3.4 LAMP-LFD sensitivity

**FIGURE 5:**
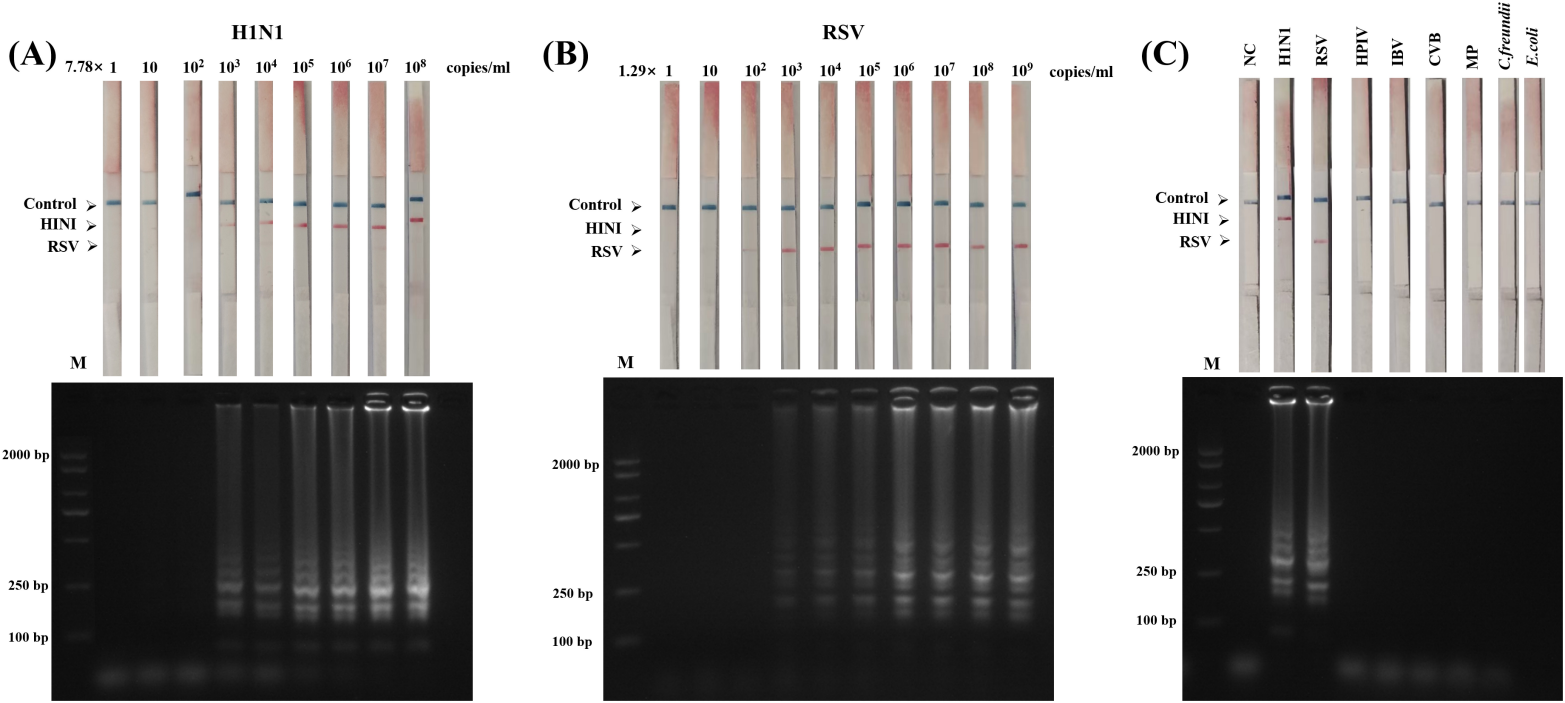
Sensitivity and specificity of the LAMP-LFD assay for H1N1 and RSV detection. **(A)** LAMP-LFD detection using serially diluted genomic DNA of H1N1 (ranging from 7.78 × 10^8^ to 1 copies/mL) as template. **(B)** LAMP-LFD detection using serially diluted genomic DNA of RSV (ranging from 1.29 × 10^9^ to 1 copies/mL) as template. **(C)** Specificity assessment: LAMP-LFD detection of six other pathogens (HPIV: human parainfluenza virus, IBVs: influenza B viruses, MP: *Mycoplasma pneumoniae*, CVB: coxsackievirus, *Citrobacter freundii*, and *E. coli*: *Escherichia coli*.) using the H1N1 and RSV detection systems. Top panel: Lateral flow device (LFD) results; Bottom panel: Agarose gel electrophoresis (AGE) results.

To determine the sensitivity of our LAMP-LFD detection method, the H1N1 and RSV templates were serially diluted 10 times, as described above. The concentration gradient of the H1N1 template was 7.78 × 10^8^–10^0^ copies/mL, and that of the RSV template was 1.29 × 10^9^–10^0^ copies/mL. At the target concentration of 7.78 × 10^3^ copies/mL, the LAMP-LFD detection results showed a clear detection line (T1 line) on the H1N1 test strip and the corresponding AGE results showed a typical ladder strip (Figure 5A), indicating that the detection limit was comparable to that of AGE. At the target concentration of 1.29 × 10^2^ copies/mL, the RSV immunochromatographic test strip showed a faint red detection line (T2 line) but AGE results did not appear. The ladder electrophoresis band (Figure 5B) indicated that the sensitivity of LFD for RSV detection is 10 times higher than that of AGE. In summary, the LAMP-LFD detection limits for H1N1 and RSV were 7.78 × 10^3^ and 1.29 × 10^2^ copies/mL, respectively.

### 3.5 LAMP-LFD specificity

To evaluate the specificity of the LAMP-LFD method, the DNA/RNA of four common respiratory pathogens (HPIV, IBV, CVB, and MP) and two other pathogens (C. freundii and *E. coli*) were used as non-target templates for cross-reactivity tests. AGE showed typical LAMP ladder bands when H1N1 and RSV genomes were used as templates, whereas no amplification bands were observed for the six non-target pathogens (Figure 5C). Similarly, LFD detection revealed that only the H1N1 and RSV samples showed clear bands on the detection line (T line; positive) while the remaining pathogens only showed bands on the quality control line (C line; negative) (Figure 5C). These results confirm that the dual LAMP-LFD method is highly specific for the target virus (H1N1 or RSV) and exhibits no cross-reactivity.

### 3.6 Clinical application of respiratory pathogen detection

**FIGURE 6:**
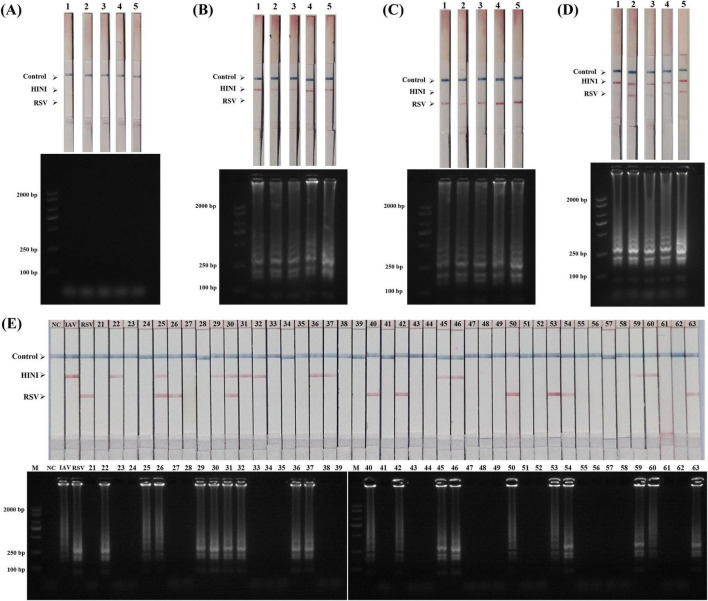
Validation of dual LAMP-LFD assay using clinical throat swab samples. **(A)** Negative results from 5 samples; **(B)** H1N1-positive results from 5 samples; **(C)** RSV-positive results from 5 samples; **(D)** H1N1and RSV positive results from 5 samples. **(E)** Other 43 clinical samples.

To verify the applicability of the developed dual LAMP-LFD detection method to real samples, 63 clinical throat swab samples (including 36 H1N1-/RSV-positive and 27 negative samples) confirmed via RT-qPCR by the First Division Hospital of Xinjiang Production and Construction Corps were used. After nucleic acids were extracted from the samples, RT-LAMP amplification was performed under optimized conditions and the amplicons were analyzed via AGE and LFD. The results are shown in Figure 6. Among the 63 clinical samples, 34 positive samples (including 15 H1N1 single positive samples, 12 RSV single positive samples and 7 co-infected positive samples) and 29 negative samples were detected by LAMP-LFD. All the negative samples detected by the LFD test strip showed a quality control line (C line) blue band. When the positive samples (including H1N1 single positive, RSV single positive and co-infected samples) were detected, the C line and the corresponding detection line (T line) were simultaneously displayed. The results of LFD and agarose gel electrophoresis were completely consistent. However, in comparison with RT-qPCR results, two H1N1-positive samples were not detected by the LAMP-LFD assay. This discrepancy may be attributed to low viral load in these samples. Despite this, as summarized in Table 2, the dual LAMP-LFD method still showed excellent specificity (100%), sensitivity (94.44%) and accuracy (96.83%) in actual clinical throat swab samples with RT-qPCR as the gold standard. There was no statistically significant difference between the two methods (*P* > 0.05). These results indicate that the LAMP-LFD detection method has good diagnostic performance, confirming the feasibility of the dual LAMP-LFD detection method for real samples.

**TABLE 2:** Comparison of LAMP-LFD detection and RT-qPCR detection of actual sample results.

Sample	Target	LAMP-LFD		RT-qPCR		Positive predictive value (%)	Negative predictive value (%)	Sensitivity (%)*	Specificity (%)*	Accuracy (%)*
		Positive	Negative	Positive	Negative					
63	H1N1	22	41	24	39	100.00	93.10	94.44	100.00	96.83
	RSV	19	44	19	44					

*Sensitivity = [TP/(TP+FN)]*100, Specificity = [TN/(TN+FP)]*100. Accuracy = [(TP+TN) (TP+TN+FN+FP)]*100. TP, true positive; FP, false positive; FN, false negative; TN, true negative.

## 4 Discussion

The influenza A (H1N1) virus and RSV are highly pathogenic respiratory pathogens that pose a serious threat to human health. The influenza A virus causes persistent antigenic drift due to high-frequency mutations in its HA gene (Morehouse et al., 2022), which significantly reduces the efficiency of antibody recognition of viral epitopes, resulting in a significant decrease in the sensitivity of traditional immunodetection methods. Although the current gold-standard method for nucleic acid detection, RT-qPCR, offers high sensitivity and specificity, its reliance on a controlled laboratory environment, specialized operational expertise, and substantial cost impedes its widespread application in resource-limited settings. Although lateral flow immunochromatographic assays (e.g., colloidal gold test strips) are easy to perform, the missed detection rate of low-viral load samples is as high as 20%–50% (Morehouse et al., 2022), especially for influenza A virus and RSV. In addition, the detection limit exceeds 10_3_ TCID50/mL (Wang et al., 2023), which does not meet the requirements for early diagnosis. Yao et al. (2023) developed a new rapid nucleic acid detection method for influenza A virus by assembling a catalytic hairpin method without enzymes and efficient isothermal amplification into colloidal gold immunochromatography. Although it can be used for the rapid and visual detection of influenza A virus, the accuracy of clinical detection only reaches 74% at room temperature. Bel Hadj Ali et al. (2024) developed a SARS-CoV-2 detection method using a one-step rapid multiplex RT-PCR combined with lateral flow immunochromatography (PCRD), which demonstrated high sensitivity (88%) and specificity (98%) in clinical samples. Although this technique eliminates the need for an RNA extraction step, the entire detection process still requires 80 min. In recent years, LAMP has become a research hotspot for the molecular diagnosis in POCT because of its constant-temperature amplification, high amplification efficiency (exponential cumulative amplification), and high specificity (Yang et al., 2024). Cheng et al. (2011) developed a single RT-LAMP assay for the detection of H1N1 with a sensitivity of 10 copies/reaction, but it relies on the results of gel electrophoresis, which is prone to aerosol pollution. LAMP is also more likely to produce non-specific amplification and primer dimers because the number of primers is far greater than that of other nucleic acids (Kim et al., 2023), making detection using methods that rely on distinguishing changes in fluorescence or pH difficult (Selva Sharma and Lee, 2024). In response to this problem, nucleic acid test strips based on lateral flow chromatography technology, which modify specific primers and design special probes, can significantly improve the specificity of the LAMP assay. Baksh et al. (2021) proposed a one-pot RT-LAMP detection strategy based on a lateral flow assay (LFA), in which viral lysis is directly performed using NaOH to release RNA, enabling rapid detection of SARS-CoV-2 (LoD = 2 copies/μL) via isothermal amplification and visual readout on the LFA. This method completes the entire process from sample collection to result output within 40 min, without requiring RNA extraction steps or specialized equipment, significantly reducing both the operational threshold and time cost. Clinical validation demonstrated 100% consistency with qPCR results and no cross-reactivity. On the other hand, multiple LAMP (m-LAMP) technology has also made significant progress. Guo J. et al. (2024) developed a multiplex LAMP detection platform that integrates a dual-labeled probe system with an LFA to achieve simultaneous detection of hepatitis B and C viruses (HBV and HCV, respectively) using a single test tube and a single test strip within 30 min. This method exhibited very high sensitivity (HBV, 10 copies/reaction; HCV, 1000 copies/reaction). Park et al. (2021) proposed a cascade amplification strategy. The target sequence was pre-amplified using RT-RPA or RT-LAMP, and the CRISPR-Cas12 system was used to specifically recognize the influenza A and B viruses. Cas12 can non-specifically cleave reporter molecules after activation, resulting in cascade signal amplification. The detection limit reached 1 × 100 colony-forming units/reaction influenza A and B virus titers, significantly improving specificity and preventing cross-reactivity. This design significantly overcame the problem of false-positives caused by primer dimer formation in traditional LAMP (Jang et al., 2020).

Although our developed duplex LAMP-LFD assay enables simultaneous detection of IAV and RSV nucleic acids within 40 min, it still relies on an additional nucleic acid extraction step for clinical samples, which remains an obstacle to achieving truly simplified and instrument-free rapid on-site testing. It is noteworthy that the field of molecular diagnostics is rapidly advancing toward fully integrated solutions. Several pioneering platforms have successfully demonstrated the feasibility of sample-to-result detection without the need for separate nucleic acid extraction, while also incorporating effective contamination control measures. For instance, Tang et al. (2022) developed a saliva-based self-testing mobile platform (SLIDE) utilizing RT-LAMP, which integrated thermal lysis pretreatment, microfluidic auto-partitioning, and real-time fluorescence detection to achieve highly sensitive detection of SARS-CoV-2, with a limit of detection (LOD) of 5 copies/μL. This platform completes the entire process from sample collection to result output in just 45 min, without requiring RNA extraction or complex operations, thereby significantly lowering the user threshold. Similarly, Simms et al. (2023) developed and evaluated the Lucira Check-It COVID-19 point-of-care test (NAAT-RDT), based on RT-LAMP, which targets the N gene regions of SARS-CoV-2 to enable rapid molecular detection within 10 to 30 min. This method eliminates the need for RNA extraction or sophisticated instrumentation, and clinical validation demonstrated high sensitivity (92.9%) and specificity (98.3%). Its portability and operational simplicity significantly enhance detection efficiency, making it suitable for large-scale screening in high-risk settings and offering a practical solution to reduce laboratory testing burdens and improve public health response coordination. Iglesias-Ussel et al. (2025) developed the Abbott ID NOW™ COVID-19 2.0 rapid molecular detection platform based on NEAR isothermal amplification technology, which targets the RdRp gene of SARS-CoV-2 to enable rapid detection (≤ 12 min) directly from swab samples. This assay demonstrated high sensitivity (PPA = 91.7%) and specificity (NPA = 98.4%) in symptomatic subjects, significantly reduces the detection time, and eliminates the need for an RNA extraction step. It thereby serves as an efficient tool for rapid diagnosis of acutely symptomatic patients and for guiding optimal timing of antiviral therapy. Meanwhile, the integration of microfluidic technology with LAMP amplification is becoming increasingly mature, advancing molecular diagnostics toward automation, multiplexing, and system integration. This combination offers an efficient and low-contamination solution suitable for multiplex POCT of influenza viruses in resource-limited settings. Sun et al. (2024) developed an integrated microfluidic nucleic acid testing platform that performs rapid RNA extraction via a magnetic bead-based method (within 3 min) coupled with dual-mode amplification using either RT-qPCR or RT-LAMP. The platform employs a PMMA microfluidic chip that integrates sample processing and reaction units, completing the entire process within 28 min at a cost as low as $9.5 per test. It achieves high-sensitivity detection of SARS-CoV-2, with a detection limit of < 297 copies, thereby significantly reducing the risk of false negatives caused by viral mutations. However, the platform exhibits relatively severe nucleic acid extraction loss. In a separate study, Zhao et al. (2025) designed a four-channel centrifugal microfluidic chip based on RT-LAMP, which enables highly sensitive (detection limit as low as 10^2^ copies/μL) and specific detection of influenza A virus and influenza B Victoria lineage. The chip utilizes centrifugal force to drive sample distribution and mix with pre-loaded primers, achieving isothermal amplification within 40 min and enabling real-time fluorescence-based result interpretation. The closed-tube design effectively prevents aerosol contamination. Compared to conventional RT-PCR, this method shortens the detection time to under one hour and supports parallel testing of eight samples, greatly enhancing screening efficiency.

In this study, a dual-labeled probe system (H1N1: DIG/biotin; RSV: FAM/biotin) was developed to achieve the simultaneous and specific recognition of influenza A virus H1N1 and RSV on a single test strip. Compared with traditional nucleic acid detection, the entire detection time can be reduced to 40 min with good sensitivity and specificity. Additionally, The use of two-color latex microspheres establishes a clearer and more intuitive visual interpretation system for multiplex detection, thereby reducing the risk of misinterpretation during point-of-care testing (POCT), no special equipment is needed, and the results can be visually detected according to the ribbon on the test strip. By optimizing the LAMP and LFD reaction systems, the LOD of the established visual LAMP-LFD detection method for H1N1 influenza virus and RSV can reach 7.78 × 10^3^ and 1.29 × 10^2^ copies/mL, which is the same as that of the gold standard for nucleic acid detection, RT-qPCR. However, it has obvious advantages in the actual operating environment. Nevertheless, we need to objectively acknowledge that this study has certain limitations in LOD assessment: due to constraints in experimental resources, we were unable to obtain appropriate viral reference materials, so the reported sensitivity results are all based on purified plasmid DNA; in addition, for the determination of extremely low copy concentration samples, we only used the 10-fold serial dilution method, and did not perform absolute quantification through more accurate techniques such as digital PCR (dPCR), which may introduce certain uncertainty to the accuracy of the extremely low LOD results. Although the LAMP-LFD detection method we established using DNA as a template, the clinical validation results still demonstrated excellent diagnostic performance. Clinical sample verification showed that the consistency between the detection and RT-qPCR results was 96.83%, which was significantly better than traditional antigen detection. This detection method provides an efficient solution for the simultaneous visual detection of influenza A virus and RSV in resource-limited environments and has practical application value for clinical diagnosis.

## Data Availability

The authors acknowledge that the data presented in this study must be deposited and made publicly available in an acceptable repository, prior to publication. Frontiers cannot accept a manuscript that does not adhere to our open data policies.

## Footnotes

1
https://www.ncbi.nlm.nih.gov/

2
https://primerexplorer.eiken.co.jp/

3
https://www.nupack.org
