# Supplementary material for: A rapid and visual dual LAMP-LFD assay for on-site simultaneous detection of influenza A virus (H1N1) and respiratory syncytial virus (RSV)
Source: Front Microbiol. 2025 Nov 10;16:1678396. doi: 10.3389/fmicb.2025.1678396 (PMC12640905; doi:10.3389/fmicb.2025.1678396)
Supplement: Supplementary file 1 [file Data_Sheet_1.docx]

Supplementary Material

## Supplementary Figures


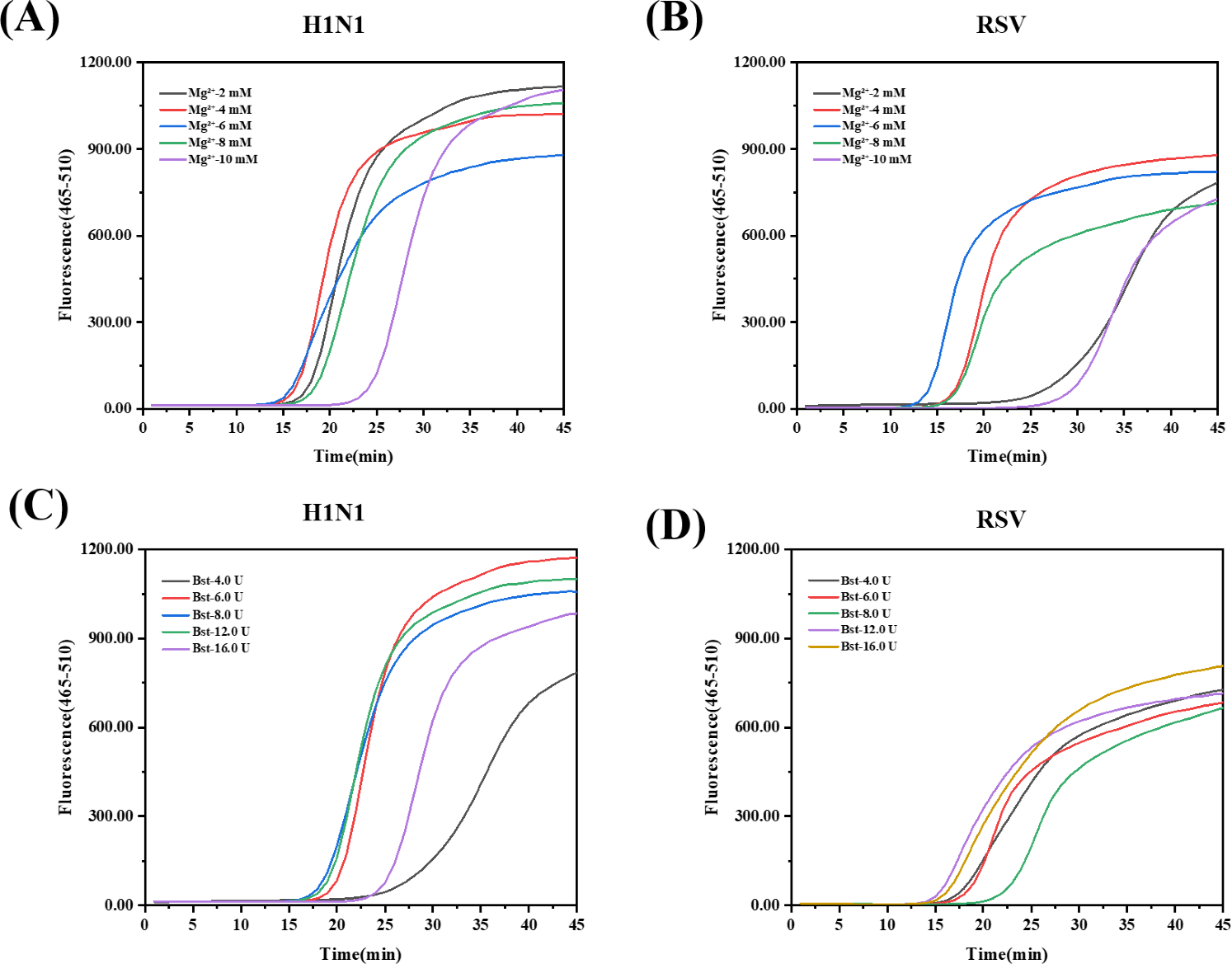


Supplementary Figure S1. Real-time fluorescence curves of LAMP amplification under varied conditions. (A) H1N1 with gradient Mg²⁺ concentrations; (B) RSV with gradient Mg²⁺ concentrations; (C) H1N1 with different Bst DNA polymerase activities; (D) RSV with different Bst DNA polymerase activities.


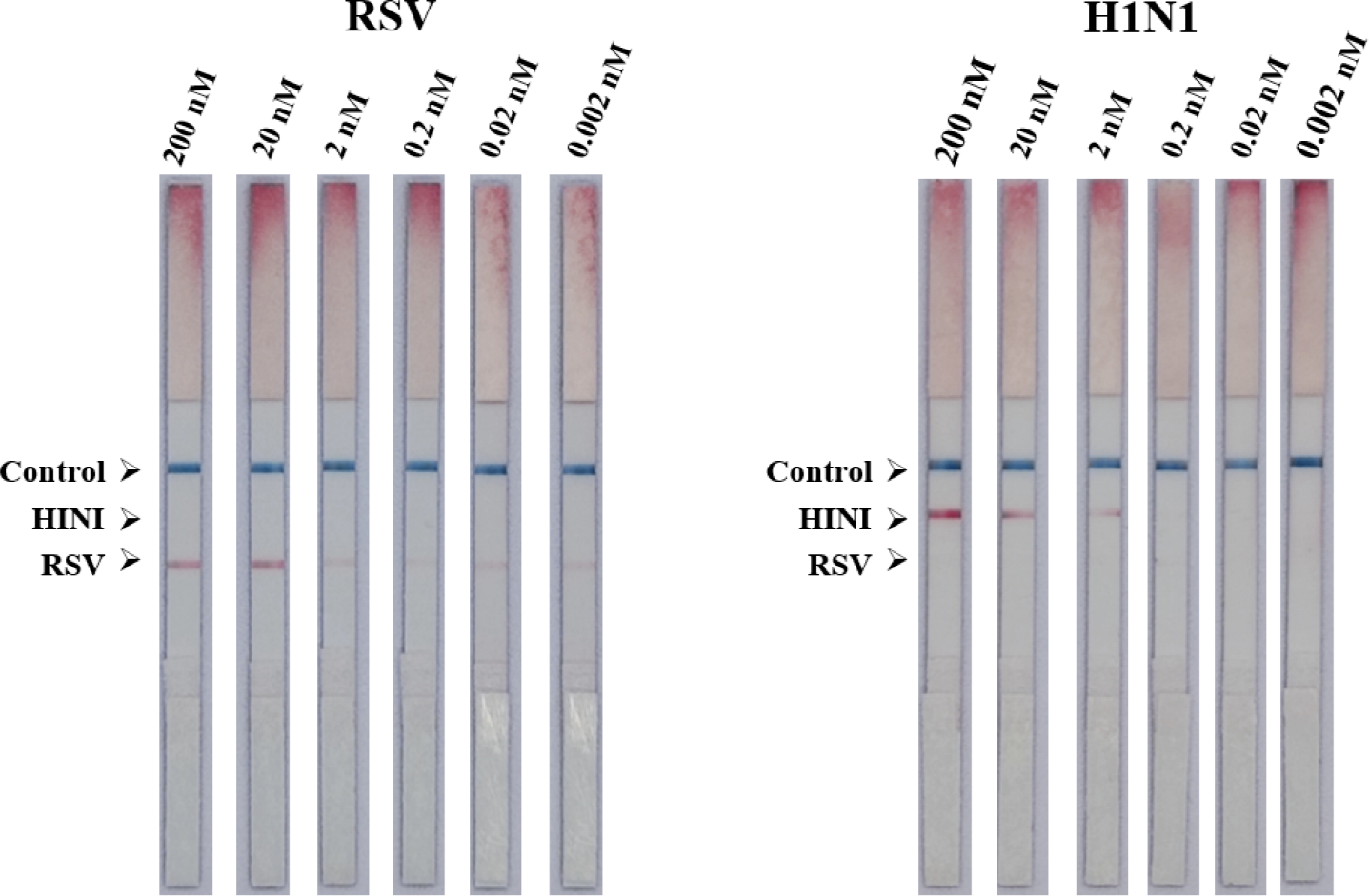


Supplementary Figure S2. Probe hybridization optimization for H1N1 and RSV detection via lateral flow dipstick. Strips show detection results for H1N1 (right) and RSV (left) across a concentration range of 200, 20, 2, 0.2, 0.02, and 0.002 nM.
